# Eating habits and behaviors in children with Dravet syndrome: A case–control study

**DOI:** 10.1111/epi.18179

**Published:** 2024-11-06

**Authors:** Alexandra Laliberté, Lyna Siafa, Arij Soufi, Christelle Dassi, Sophie J. Russ‐Hall, Ingrid E. Scheffer, Kenneth A. Myers

**Affiliations:** ^1^ Faculty of Medicine and Health Sciences McGill University Montreal Quebec Canada; ^2^ Research Institute of the McGill University Medical Centre Montreal Quebec Canada; ^3^ Epilepsy Research Centre, Department of Medicine (Austin Health) The University of Melbourne Heidelberg Victoria Australia; ^4^ Bladin‐Berkovic Comprehensive Epilepsy Program, Department of Neurology Austin Health Heidelberg Victoria Australia; ^5^ Florey Institute of Neuroscience and Mental Health Melbourne Victoria Australia; ^6^ Murdoch Children's Research Institute and Department of Paediatrics University of Melbourne, Royal Children's Hospital Melbourne Victoria Australia; ^7^ Division of Neurology, Department of Pediatrics, Montreal Children's Hospital McGill University Health Centre Montreal Quebec Canada; ^8^ Department of Neurology and Neurosurgery McGill University Health Centre Montreal Quebec Canada

**Keywords:** anorexia, Dravet syndrome, dysphagia, eating, feeding, gastrostomy

## Abstract

This study evaluated food preferences and eating behaviors of individuals with Dravet syndrome. Patients diagnosed with Dravet syndrome were recruited, as well as a control group composed of siblings of patients with epilepsy (any form). The Food Preference Questionnaire and the Child Eating Behavior Questionnaire were completed by caregivers along with two open‐ended questions regarding eating challenges. Seventy‐eight participants (45 with Dravet syndrome and 33 controls) were included. Compared to controls, mean scores for food preference were lower for fruits (*p* = .000099), meats and fish (*p* = .00094), and snacks (*p* = .000027) in Dravet syndrome. People with Dravet syndrome also had less emotional overeating (*p* = .0085) and food enjoyment (*p* = .0012), but more slowness in eating (*p* = .00021) and food fussiness (*p* = .0064). In a subgroup analysis of only pediatric (age <18 years) patients, similar results were observed for both food preferences and eating habits. In qualitative data, caregivers most commonly reported difficulties with fixation on specific foods. This study demonstrates specific food preferences and challenging eating behaviors in individuals with Dravet syndrome. These data provide potential avenues for nutritional interventions and behavioral therapies to increase the quality of life of patients and their families.

## 1 INTRODUCTION

Dravet syndrome is an infantile‐onset developmental and epileptic encephalopathy (DEE) involving febrile and afebrile seizures, with onset at a median age of 6 months.^1^, ^2^ More than 80% of patients have heterozygous pathogenic variants in *SCN1A* (OMIM 182389), of which ~90% occur de novo.^3^ Although drug‐resistant seizures are a major concern for people with Dravet syndrome and their families, the significance of a vast array of morbidities is increasingly recognized as critical influences that affect the quality of life of patients and their families. In addition to developmental impairment and intellectual disability, people with Dravet syndrome have an increased risk for autism spectrum disorder, cardiovascular problems, dysautonomia, gait abnormalities, sleep disturbances, and sudden unexpected death in epilepsy.^4^, ^5^, ^6^, ^7^ Challenges with feeding have also been reported by caregivers, which may affect quality of life for both the individual and their family^8^; however, the precise nature of eating habits and feeding behaviors in people with Dravet syndrome has not been thoroughly examined.^4^ In this study, we evaluated the food preferences and eating behaviors of people with Dravet syndrome, and compared them to a healthy control cohort.

## 2 METHODS

### 2.1 Participants

Patients with Dravet syndrome were recruited from the neurology clinics at Montreal Children's Hospital (Montreal, Quebec, Canada) and the Dravet Clinic at Austin Health (Heidelberg, Victoria, Australia) between January 2021 and January 2023. Caregivers were invited to complete a questionnaire package comprising (1) a Food Preference Questionnaire (FPQ)^9^; (2) a Child Eating Behavior Questionnaire (CEBQ)^10^; (3) two additional free text questions to characterize particular feeding challenges families have experienced with their children (“*Can you describe any particular challenges you've experienced in feeding your child?*” and “*If you have had difficulties with feeding your child, at what age did they start, and at what age were they most severe (challenging)?*”); and (4) self‐reported age, height, and weight. In addition, we recruited a control group of healthy siblings of patients with epilepsy (any form) from the Montreal Children's Hospital Neurology Clinic, and asked caregivers to complete the same questionnaire package. Caregivers had the option to complete the questionnaires in English or French.

### 2.2 Questionnaires

The Food Preference Questionnaire (or FPQ) is a validated 75‐food item parent‐reported questionnaire that assesses food preferences in children.^9^ Food preferences are assessed based on six food groups and their associated food items: vegetables (19 food items), fruits (16 food items), meat and fish (13 food items), dairy (9 food items), snacks (12 food items), and starch (6 food items). Caregivers were asked to rate their child's preferences on a 5‐point scale: −2 = dislikes a lot, −1 = dislikes, 0 = neither dislikes or likes, 1 = likes, and 2 = likes a lot; there is also an option to choose “has never tried,” in which case the specific food item is not scored. For each food category, a total score was calculated based on individual scoring given to food items and divided by the total food items within the food category. Food items without responses were subtracted from the total food items within the food category.

The Child Eating Behavior Questionnaire (or CEBQ) is a 35‐item questionnaire that assesses feeding patterns in children.^10^ Eating behavior is assessed through eight feeding dimensions: food responsiveness (four items), enjoyment of food (four items), emotional overeating (four items), desire to drink (three items), satiety responsiveness (five items), slowness in eating (four items), emotional undereating (four items), and fussiness (seven items). Parents were asked to rate the frequency of their child's behaviors on a 5‐point scale: 1 = never, 2 = rarely, 3 = sometimes, 4 = often, and 5 = always. For five questions, the scale point was reversed: 1 = always, 2 = often, 3 = sometimes, 4 = rarely, and 5 = never.

Statistical comparisons of food preferences and child's eating behavior between individuals with Dravet syndrome and the control group were made using the Mann–Whitney test and generalized linear model with gamma approximation and log‐link function. A subgroup analysis was performed, comparing only the pediatric participants (defined as age <18 years). The Holm‐Bonferroni method was used to correct for multiple comparisons. Statistics were performed using IBM SPSS Statistics (version 29.0.1.1).

This study was approved by the McGill University Heath Centre Research Ethics Board (2018‐3937) and the Human Research Ethics Committee of Austin Health (H2007/02961). Written informed consent was obtained from participants or caregivers.

## 3 RESULTS

### 3.1 Demographics

Sixty‐nine patients with Dravet syndrome were approached to participate, of whom 45 (34 <18 years of age) agreed to participate and had data included (Table 1). An additional 33 healthy controls (30 <18 years of age), were recruited into the study. Of the patients with Dravet syndrome, eight were diagnosed with autism spectrum disorder, and an additional seven had “autistic traits” or “autistic features” noted in their charts. Mean age ± standard deviation (SD) for the patients with Dravet syndrome was 12.0 ± 10.6 years and for controls was 10.2 ± 5.0 years. When considering only the participants younger than 18 years of age, the Dravet group was slightly younger, with mean age 7.0 ± 5.2 years vs 9.2 ± 3.9 years for the controls. There was no significant difference in body mass index between the groups. For one individual with Dravet syndrome, both parents filled out the questionnaires independently and both sets of scores were included.

**TABLE 1:** Demographics and Data From Food Preference and Eating Behavior Questionnaires.

	DS (total) (*n* = 45)	Control (total) (*n* = 33)	*p*	DS (<18 y) (*n* = 34)	Control (<18 y) (*n* = 30)	*p*
Sex						
Male	18	17		14	15	
Female	27	16		20	15	
Age in years (mean ± SD)	12.0 ± 10.6	10.2 ± 5.0		7.0 ± 5.2	9.2 ± 3.9	
BMI (mean ± SD (kg/m^2^)*	19.7 ± 6.1	19.6 ± 4.8	.93	18.8 ± 6.4	18.6 ± 4.0	.81
Food preferences						
Vegetables	.28 ± .74	.54 ± .62	.064	.22 ± .77	.51 ± .63	.073
Fruits	.50 ± .80	1.17 ± .57	9.9 × 10^−5^	.52 ± .82	1.19 ± .59	4.3 × 10^−4^
Meats and fish	.44 ± .83	1.00 ± .58	9.4 × 10^−4^	.34 ± .89	.99 ± .59	1.5 × 10^−3^
Dairy	.83 ± .73	.82 ± .49	.75	.85 ± .80	.88 ± .44	.94
Snacks	.60 ± .78	1.33 ± .49	2.7 × 10^−5^	.67 ± .76	1.33 ± .49	2.5 × 10^−4^
Starches	.68 ± .52	.85 ± .46	.17	.71 ± .50	.86 ± .45	.31
Eating behavior						
Food responsiveness	2.19 ± .94	2.36 ± .79	.22	2.24 ± .88	2.39 ± .77	.36
Emotional overeating	1.54 ± .60	1.95 ± .73	8.5 × 10^−3^	1.50 ± .55	1.92 ± .64	9.4 × 10^−3^
Enjoyment of food	3.31 ± 1.00	4.00 ± .65	1.2 × 10^−3^	3.31 ± 1.03	3.99 ± .64	4.2 × 10^−3^
Desire to drink	2.44 ± 1.18	2.63 ± .96	.30	2.50 ± 1.20	2.66 ± .92	.39
Satiety responsiveness	2.88 ± .84	2.74 ± .57	.33	2.93 ± .89	2.75 ± .58	.28
Slowness in eating	3.21 ± .93	2.42 ± .71	2.1 × 10^−4^	3.33 ± .95	2.42 ± .68	1.7 × 10^−4^
Emotional undereating	3.01 ± 1.13	2.87 ± .73	.53	3.18 ± 1.14	2.87 ± .75	.23
Food fussiness	3.21 ± 1.15	2.51 ± .91	6.4 × 10^−3^	3.31 ± 1.17	2.52 ± .94	6.1 × 10^−3^

* *BMI data were available for only 31 Dravet syndrome patients (23 <18 years of age) and 27 controls (24 <18 years of age). The Holm‐Bonferroni method was used to correct for multiple comparisons. Significant differences are set in bold and italics. BMI, body mass index; DS, Dravet syndrome; SD, standard deviation.

### 3.2 Questionnaires

Mean scores and associations for food preferences and children's eating behaviors between the Dravet syndrome and control groups are shown in Table 1. Food preference scores were lower in the Dravet syndrome group for fruits (*p* = .000099), meats and fish (*p* = .00094), and snacks (*p* < .000027). As shown in Table 2, participants with Dravet syndrome are 60% less likely to consume fruits, 66% less likely to eat meat and fish, and nearly 55% less likely to consume snacks.

**TABLE 2:** Dravet syndrome associations with specific feeding patterns.

	RR	95% CI	*p*
Food preference scores			
Vegetables	.43	.13–1.45	.173
Fruits	.41	.22–.76	.0004
Meats and fish	.34	.14–.84	.02
Dairy	1.01	.70–1.45	.957
Snacks	.45	.26–.78	.005
Starches	.82	.59–1.15	.256
Child eating behavior scores			
Food responsiveness	.91	.77–1.09	.322
Emotional overeating	.76	.65–.90	.001
Enjoyment of food	.82	.73–.92	.001
Desire to drink	.95	.77–1.16	.612
Satiety responsiveness	1.05	.94–1.18	.361
Slowness in eating	1.33	1.18–1.51	<.0001
Emotional undereating	1.03	.89–1.20	.44
Food fussiness	1.3	1.14–1.50	.001

Generalized linear model with gamma approximation and log‐link function was used. Regression betas were expressed as risk ratios (RRs). CI, confidence interval.

With respect to eating behaviors, patients with Dravet syndrome were 24% less likely to have emotional overeating (*p* = .0085) and had 18% less food enjoyment (*p* = .0012) compared with controls. Patients with Dravet syndrome were also 30% more likely to have slowness in eating (*p* = .00021) and food fussiness (*p* = .0064).

When comparing the associations in the pediatric (age <18 years) patients only, the findings were similar. Children with Dravet syndrome were less likely to consume fruits (*p* = .00043), meats and fish (*p* = .0015), and snacks (*p* = .00025), and less likely to have emotional overeating (*p* = .0094) and food enjoyment (*p* = .0042) than children in the control group. The pediatric patients with Dravet syndrome were also more likely to have slowness in eating (*p* = .00017) and food fussiness (*p* = .0061) compared to the pediatric controls.

### 3.3 Qualitative data

When asked about challenges faced regarding food preferences and eating behaviors, 39 of 45 caregivers responded to the free text questions for individuals with Dravet syndrome and 20 of 33 for the controls. For the Dravet syndrome group, the most commonly identified challenges included food fixation (9 comments), limited variety in food (8), picky eating (7), difficulty with textures (5), and loss of appetite attributed to medication side effects (7). The age at which the eating challenges began ranged from 6 months (mostly at the usual age for initiation of solid food) to 15 years. Although the vast majority of caregivers of controls reported no feeding difficulties, fluctuation in food interest and picky eating were the main challenges noted, with age at onset ranging from less than 1 year to 9 years.

Some examples of comments from Dravet syndrome caregivers included: “*Was eating everything until 3 years of age, then became very selective … Between 3* and *4 years of age approximately, he only ate yogurt*.” and “*Will only eat meat and veggies 99.9% of the time. Same foods every day, same breakfast every day*.” and “*Eats the exact same food, at the exact same time every day. So, no matter where we are or where we go, I have to have her food with me*.”

## 4 DISCUSSION

Our study highlights the major difficulties in feeding observed in people with Dravet syndrome. This population tends to have specific food preferences, as well as eating behaviors, that pose problems for the individual and their caregivers. Our findings show that individuals with Dravet syndrome have reduced preferences for specific food categories, namely fruits, meats and fishes, and snacks; however, they were similar to controls in their affinity for dairy and starches. The eating behavior questionnaire data found that people with Dravet syndrome derive less enjoyment from eating, eat more slowly, and are fussier than healthy controls. Our qualitative data reinforced these findings, and also highlighted that people with Dravet syndrome can become highly fixated on specific foods, sometimes refusing all but a few specific dishes.

Understanding feeding difficulties in Dravet syndrome is extremely important, as this morbidity can severely impact the quality of life of the patient and their family. The rate of gastrostomy tube insertion in people with Dravet syndrome is significant, but varies notably across regions and cultures, being as high as 17%–18% in British and Dutch data, but only ~2% in the Austin Health Dravet clinic cohort (unpublished data).^11^, ^12^, ^13^ The need for such interventions might be reduced if more targeted strategies for improving oral feeding could be developed.

Our results complement published data on feeding in Dravet syndrome. Minderhoud and co‐authors conducted a study regarding gastrointestinal and eating problems in individuals with *SCN1A*‐related epilepsies, the majority of whom had Dravet syndrome.^11^ They found high rates of drooling, distraction during mealtime, constipation, and loss of appetite. A study by Clayton et al. regarding gastrostomy and feeding difficulties in people with Dravet syndrome in the United Kingdom found that feeding difficulties most commonly involved being picky/fussy and having low appetite.^13^ A survey done by Knupp and colleagues provided more details to how feeding difficulties were experienced by caregivers. In their large survey, they found that 99% of caregivers of children with Dravet syndrome identified at least one appetite symptom and reported eating difficulties included eating a lower variety of food, prolonged mealtime, and picky eating.^8^

Our study is not without limitations. First, the CEBQ and FPQ are tools that have been designed for a pediatric population. However, in a cohort of individuals with intellectual disability, requiring the support of caregivers, we deemed it appropriate to use these questionnaires in our adult participants as well. Our similar findings between the pediatric and adult patients with Dravet syndrome suggest that this was a valid approach. In addition, although the mean ages were similar between our Dravet syndrome and control groups, we were not able to do precise age‐matching, and this may affect the reliability of our results. Autism had been diagnosed in 22% of individuals in the Dravet group, consistent with what has been reported previously.^14^, ^15^ As patients with autism often have feeding difficulties,^16^ this could be a confounding factor. An additional possible source of bias was that food preference data were collected primarily from caregivers' based on their interpretations of their children's behaviors, rather than the individuals themselves who were completing the surveys.

In conclusion, we showed that food selectivity is common in individuals with Dravet syndrome, and that specific eating behaviors are more prominent, primarily food fussiness, slowness in eating, and decreased food enjoyment. To address feeding difficulties in people with Dravet syndrome, a complete nutritional assessment is recommended with careful monitoring of growth, and consideration of psychological therapies to help address problematic feeding behaviors before moving to gastrostomy insertion. Further studies exploring the reasons behind cultural and regional differences in insertion of gastrostomies in patients with Dravet syndrome and DEEs more broadly would also inform management strategies and ensure that such decisions facilitate optimization of quality of life for patients and their families. Support to caregivers and families should also be offered by the treating team to help ease the burden of feeding difficulties in the context of a disease that is already very challenging to manage.^17^

## AUTHOR CONTRIBUTIONS

Laliberté assisted with data collection, analyzed data, and wrote the initial draft of the manuscript. Siafa assisted with study design and data collection. Soufi assisted with data collection. Dassi assisted with data collection. Russ‐Hall assisted with data collection. Scheffer assisted with data collection and reviewed and revised the manuscript. Myers conceptualized and designed the study, assisted with data analysis, and reviewed and revised the manuscript.

## FUNDING INFORMATION

This study was supported by funding from Fonds de Recherche du Québec—Santé (282228, 295639) and the National Health and Medical Research Council of Australia (GNT1091593, GNT1172897, GNT2006841).

## CONFLICT OF INTEREST STATEMENT

Dr. Myers has received research funding support from Dravet Canada and is on an advisory board for Jazz Pharmaceuticals. Prof. Scheffer has served on scientific advisory boards for BioMarin, Chiesi, Eisai, Encoded Therapeutics, GlaxoSmithKline, Knopp Biosciences, Nutricia, Rogcon, Takeda Pharmaceuticals, UCB, Xenon Pharmaceuticals, Cerecin, and Longboard Pharmaceuticals; has received speaker honoraria from GlaxoSmithKline, UCB, BioMarin, Biocodex, Chiesi, Liva Nova, Nutricia, Zuellig Pharma, Stoke Therapeutics, Eisai, and Akumentis; has received funding for travel from UCB, Biocodex, GlaxoSmithKline, Biomarin, Encoded Therapeutics, Stoke Therapeutics, and Eisai; has served as an investigator for Anavex Life Sciences, Cerevel Therapeutics, Eisai, Encoded Therapeutics, EpiMinder Inc., Epygenyx, ES‐Therapeutics, GW Pharma, Marinus, Neurocrine BioSciences, Ovid Therapeutics, SK Life Science, Takeda Pharmaceuticals, UCB, Ultragenyx, Xenon Pharmaceuticals, Zogenix, and Zynerba; has consulted for Care Beyond Diagnosis, Epilepsy Consortium, Atheneum Partners, Ovid Therapeutics, UCB, Zynerba Pharmaceuticals, BioMarin, Encoded Therapeutics, and Biohaven Pharmaceuticals; and is a Non‐Executive Director of Bellberry Ltd. and a Director of the Australian Academy of Health and Medical Sciences and the Royal Society (Australia). She may accrue future revenue on pending patent WO61/010176 (filed: 2008): Therapeutic Compound; has a patent for *SCN1A* testing held by Bionomics Inc. and licensed to various diagnostic companies; and has a patent molecular diagnostic/theragnostic target for benign familial infantile epilepsy (BFIE) [PRRT2] 2011904493 and 2 012 900 190 and PCT/AU2012/001321 (TECH ID:2012–009). The remaining authors have no conflicts of interest.

## ETHICS STATEMENT

This study was approved by the McGill University Heath Centre Research Ethics Board (2018‐3937) and the Human Research Ethics Committee of Austin Health (H2007/02961). We confirm that we have read the Journal's position on issues involved in ethical publication and affirm that this report is consistent with those guidelines.

## CONSENT

Written informed consent was obtained from participants or caregivers.

## Data Availability

The data that support the findings of this study are available from the corresponding author upon reasonable request.
